# Oral Cnm*-*positive *Streptococcus Mutans* Expressing Collagen Binding Activity is a Risk Factor for Cerebral Microbleeds and Cognitive Impairment

**DOI:** 10.1038/srep38561

**Published:** 2016-12-09

**Authors:** Isao Watanabe, Nagato Kuriyama, Fumitaro Miyatani, Ryota Nomura, Shuhei Naka, Kazuhiko Nakano, Masafumi Ihara, Komei Iwai, Daisuke Matsui, Etsuko Ozaki, Teruhide Koyama, Masaru Nishigaki, Toshiro Yamamoto, Aiko Tamura, Toshiki Mizuno, Kentaro Akazawa, Akihiro Takada, Kazuo Takeda, Kei Yamada, Masanori Nakagawa, Tokutaro Tanaka, Narisato Kanamura, Robert P. Friedland, Yoshiyuki Watanabe

**Affiliations:** 1 Department of Epidemiology for Community health and Medicine, Kyoto Prefectural University of Medicine, Kyoto, 6028566, Japan; 2 Department of Dental Medicine, Kyoto Prefectural University of Medicine, Kyoto, 6028566, Japan; 3 Department of Neurology, Kyoto Prefectural University of Medicine, Kyoto, 6028566, Japan; 4 Department of Pediatric Dentistry, Osaka University Graduate School of Dentistry, Osaka, 5650871, Japan; 5 Department of Stroke and Cerebrovascular Diseases, National Cerebral and Cardiovascular Center, Osaka, 5658565, Japan; 6 Department of Radiology, Kyoto Prefectural University of Medicine, Kyoto, 6028566, Japan; 7 Kyoto Industrial Health Association, Kyoto, 6048472, Japan; 8 North Medical Center, Kyoto Prefectural University of Medicine, Kyoto, 6292261, Japan; 9 Department of Neurosurgery, Seirei Hamamatsu General Hospital, Shizuoka, 4308558, Japan; 10 Department of Neurology, University of Louisville, KY, 40202, USA

## Abstract

Cerebral microbleeds (CMBs) are an important risk factor for stroke and dementia. We have shown that the collagen binding surface Cnm protein expressed on *cnm*-positive *Streptococcus mutans* is involved in the development of CMBs. However, whether the collagen binding activity of *cnm*-positive *S. mutans* is related to the nature of the CMBs or to cognitive impairment is unclear. Two-hundred seventy nine community residents (70.0 years) were examined for the presence or absence of *cnm*-positive *S. mutans* in the saliva by PCR and collagen binding activity, CMBs, and cognitive function were evaluated. Cnm-positive *S. mutans* was detected more often among subjects with CMBs (p < 0.01) than those without. The risk of CMBs was significantly higher (odds ratio = 14.3) in the group with *S. mutans* expressing collagen binding activity, as compared to the group without that finding. Deep CMBs were more frequent (67%) and cognitive function was lower among subjects with *cnm*-positive *S. mutans* expressing collagen binding activity. This work supports the role of oral health in stroke and dementia and proposes a molecular mechanism for the interaction.

Oral factors, such as periodontal disease, reduced number of remaining teeth, and systemic bacterial infection spreading from the oral cavity, have recently been reported to increase the risk of dementing disorders^1^,^2^,^3^. The sensitivity for the detection of asymptomatic cerebral microbleeds (CMBs), which are an early stage of cerebral hemorrhage^4^,^5^ has been enhanced by susceptibility-weighted imaging (SWI), a new MRI brain imaging technique. Cerebral microbleeds are attracting attention as an important predictive marker of stroke^6^,^7^,^8^. They are also known to be an independent factor for vascular cognitive impairment, and effects of CMBs have been reported to vary with the affected site^9^. However, the clinical significance of CMBs and the mechanisms of their production has not been clarified.

Recently, Nakano *et al*. have reported that intracerebral hemorrhage occurred more frequently in mice exposed to *Streptococcus mutans*, a major member of oral streptococci related to dental caries, with the *cnm* gene (*cnm*-positive *S. mutans*) than to mice exposed to the same organism lacking the gene, and suggested that exposure to *cnm*-positive *S. mutans* is a novel risk factor for cerebral hemorrhage in humans^10^. This exposure may be a new risk factor for cerebral vascular injury^11^. The collagen binding protein expressed on the surface of *S. mutans* is called the Cnm protein, and has affinity to bind to collagen, a component of dentin, and to the collagen layer of the vascular endothelium, as well as to fibrinogen^10^,^12^. *Cnm*-positive *S. mutans* has been reported to induce cerebral hemorrhage by binding to damaged sites on the vascular endothelium, and increasing the expression of the zinc-metalloproteinase matrix metalloproteinase-9 (MMP-9) which degrades the extracellular matrix^10^. Also, our previous study in humans showed that the collagen binding activity of *cnm*-positive *S. mutans* is an independent risk factor for the occurrence of CMBs and intracerebral hemorrhage^13^,^14^.

We evaluated whether *S. mutans* expressing Cnm protein with collagen binding activity is related to the location of CMBs, and how Cnm related CMBs are related to cognitive decline. We also evaluated the influence of apolipoprotein E genotype and oral health on CMB development and Cnm expressing *S. mutans* exposure.

## Materials and Methods

### Study Subjects and Period

In our original report, we included 139 subjects and found a direct relationship between Cnm-positive *S. mutans* and the existence of microbleeds^13^. We now present a larger study^15^ evaluate the association amongst Cnm-positive *S. mutans*, microbleeds and cognitive decline. Among the 469 subjects (including the first 139 subjects), 290 participants (195 males and 95 females; 70.0 ± 6.1 years of age) gave us consent for this study and underwent medical check-ups with brain MRI examinations and cognitive functional examination between 2012 and 2014. Of these subjects, 5 did not complete the questionnaire, 2 did not undergo brain MRI, and 4 were edentulous or had xerostoma and were excluded. As a result, 279 subjects (189 males and 90 females) were registered for this study.

This study was conducted according to the guidelines laid down in the Declaration of Helsinki, and was approved by the Ethical Review Board of the Kyoto Prefectural University of Medicine (approval number: G-144) and all participants provided signed informed consent.

### Investigation items concerning lifestyle and body measurement (clinical check-up)

Lifestyle habits were evaluated using a self-administered questionnaire checked by trained members of the study staff. This questionnaire consisted of items concerning medical past history, drug compliance, alcohol intake, smoking status and educational background. Hypertension was defined as having a history of hypertension and/or a systolic pressure ≥140 mmHg and/or a diastolic pressure ≥90 mmHg. Hyperlipidemia was defined as having a history of hyperlipidemia and/or showing a triglyceride ≥150 mg/dl, an HDL < 40 mg/dl and/or an LDL ≥140 mg/dl. Diabetes mellitus was defined as showing an HbA1c ≥6.5% and/or use of anti-diabetic medication. It was determined which subjects had a history of stroke/cardiovascular disease or had received treatments for those conditions. Those who were currently drinking alcohol every day were designated as the alcohol drinking group. Those who were smoking every day were designated as the smoking group. The subjects were also divided into 2 groups according to the number of years of schooling (≤12 years and >12 years). Four subjects did not answer the question concerning education. Systemic atherosclerosis was evaluated by measuring brachial-to-ankle pulse wave velocity (baPWV) (form PWV/ABI; Omron Healthcare Co., Ltd., Kyoto, Japan)^16^,^17^.

### Dental evaluations

We collected information concerning the number of remaining teeth, presence/absence of dental caries, and community periodontal index (CPI)^18^,^19^ by oral examination, which was performed by a single dentist (FM). Dental caries (+) was defined as having at least 1 tooth with dental caries. Concerning the CPI, the oral cavity of each participant was divided into 6 areas, and the pocket depth was measured in the tooth specified in each area by probing using a WHO periodontal probe. CPI were graded according to following categories; (0) no sign of disease, (1) gingival bleeding after gentle probing, (2) supra- or subgingival calculus, (3) pathologic pockets 4 or 5 mm deep, and (4) pathologic pockets ≥6 mm, and the maximum value of the 6 areas was regarded as the CPI of the subject^19^. Based on the results, the subjects were divided into those with and without pathologic pockets (CPI ≥ code 3).

### Laboratory evaluations

Peripheral blood samples were obtained from all participants. We measured high-sensitivity C-reactive protein (hs-CRP) and apolipoprotein E (APOE) genotype in addition to basic hematological and biochemical assays. Serum hs-CRP concentrations were measured using an established commercially available manufacturer’s protocol by latex coagulation nephelometry as part of the health screening check-up (Kyoto Biken Laboratories, Inc., Kyoto, JAPAN). APOE genotyping was performed by polymerase chain reaction (PCR) (Funakoshi Co., Ltd., Tokyo, Japan). Of the 279 subjects, 272 (97%) consented to APOE genotyping. APOE genotype was classified into 2 groups, according to the presence or absence of the APOE ε4 allele.

### Isolation of *S. mutans* strains and molecular biological detection of *cnm* encoding Cnm protein

We sampled oral bacteria from all subjects from saliva and plaque around the teeth. Each oral salivary specimen was streaked on Mitis-Salivarius agar containing bacitracin (MSB), a selective medium for mutans streptococcal species, and bacterial colonies were picked up. Bacterial DNA was extracted from the colonies in accordance with our previously reported methods and stored as DNA extracts^13^,^20^. The DNA extracts were subjected to PCR for confirming *S. mutans* using *S. mutans*-specifics primers^21^. Using *cnm*-specific primers, the presence or absence of the *cnm* gene in the *S. mutans* isolates was determined. Details of these assay methods are described in supplementary materials.

### Measurement of the Cnm protein-binding activity of *cnm*-positive *S. mutans*

We performed a collagen-binding assay to determine the collagen-binding activity of the *cnm*-positive *S. mutans* isolated from the subjects using methods previously reported^13^,^20^. For analysis of Cnm protein-binding activity, standard strains (SA83 and SA137) with binding activity of 100% was prepared, and the binding activity of the sample was expressed as the rate relative to the activity of the standard sample. The Cnm protein-binding activity was judged to be positive when this rate was 10% or higher. Details of these assay methods are described in supplementary materials.

### Evaluation of brain MRI

Brain MRI was performed using a 1.5-T scanner (Achieva 1.T, Philips N.V., Best, the Netherlands). Images were evaluated by neurologists (NK, AT) and a radiologist (KA), who are board certified specialists. Transverse T1-weighted (repetition time [TR]: 611 ms, echo time [TE]: 13 ms) and T2-weighted (TR: 4431 ms, TE: 100 ms) images obtained at 5-mm slice thickness were evaluated.

Using FLAIR and T2-weighted imaging, leukoaraiosis was categorized according to the Fazekas scale by semi-quantitative evaluation of periventricular hyperintensity (PVH) and deep white mater hyperintensity (DWMH)^22^. PVH was graded as (0) absence, (1) “caps” or pencil-thin linings, (2) halos, or (3) irregular PVH extending into deep white matter. In addition, DWMH was graded as (0) absence, (1) solitary, (2) the beginning aggregation of foci, or (3) large confluent areas of WMH^15^,^23^.

**Figure 1:**
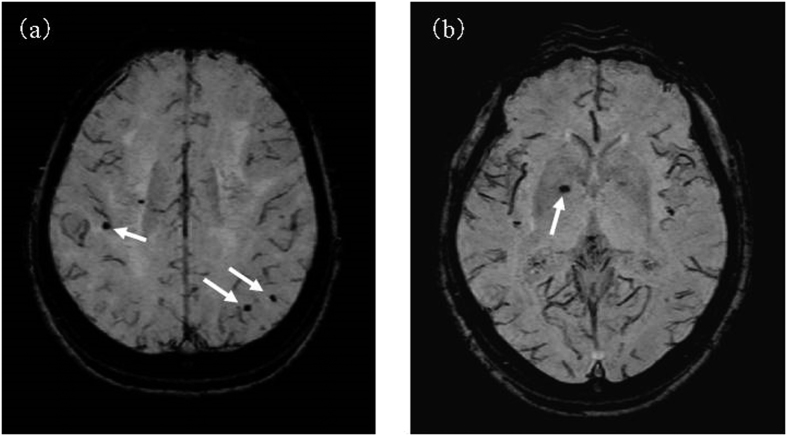
Cerebral microbleeds (arrows) using the SWI-MRI-based classification is shown in this figure. (**a**) Lobar microbleeds distributed across the temporal lobes. (**b**) Isolated deep microbleeds in the internal aspect of the right putamen.

CMBs were evaluated by susceptibility-weighted imaging (SWI) (3d-Tlffe 4 mmTHK/Gapless) of brain MRI. By SWI, CMBs can be detected because hemosiderin present in the foci of CMBs is visualized as punctate and round hypointense areas^24^,^25^. A representative MRI of microbleeds based on the SWI image is shown in Fig. 1. As shown in the figure, microbleeds were assessed based on susceptibility-weighted imaging (SWI) of brain MRI. We classified CMBs into deep, lobar, and mixed types^26^. This protocol is the same as that employed in our previous clinical MRI studies^13^,^27^. The absence of a large cerebral hemorrhage, pathological brain atrophy, or other findings suggesting an infectious disease or neurodegenerative condition was established in all subjects. We carefully performed all the MR evaluations blinded to information about the Cnm-related data. The examiners (a radiologist and neurologist) discussed the findings to obtain a consensus in case of discordant MR evaluations. Because CMBs may be confused with other signals such as blood vessels or calcifications, we carefully evaluated whether or not the findings represent CMBs using axial and sagittal scanning. Along with the total-grading score on the Fazekas classification and CMBs, the intraclass correlation coefficient (ICC) was calculated as an index of inter-rater reliability; a high ICC of 0.92 and 0.93 were obtained for both DWMLs and PVH. Thus, the data obtained had a favorable inter-rater reliability, which suggested that this grading was sufficiently reliable to be used as an accurate grading tool.

### Cognitive function evaluations

We used the Mini-Mental State Examination (MMSE)^28^ and letter fluency tasks^29^,^30^. Specifically, the subjects are instructed to orally recall as many words that start with a specified letter during a 1-minute period, and the words are counted. In this study, we counted Japanese words that start with “Ta” and “Ka”^31^. These tests of cognitive function were performed on the same day as brain MRI, and the results were recorded by trained neurologists and neuropsychologists.

### Analytical methods

The results of each factor examined were compared between sexes and according to the presence or absence of CMBs, *cnm*-positive *S. mutans*, and collagen binding activity. These group-wise comparisons were performed using the t-test and χ^2^-test. We also evaluated the risk of CMBs by multivariate analysis using sex, age, lifestyle, and the results of body measurement, oral examination, blood tests, and brain MRI as adjustment factors. These analyses were performed at the 0.05 level of significance using SPSS 19.0 J for Windows (SPSS Japan Inc., Tokyo, Japan).

## Results

### Characteristic of the subjects

Table 1 shows the characteristics of the subjects. The mean age was 70.0 ± 6.1 years, BMI was 22.7 ± 3.0 kg/m^2^, and MMSE score was 28.3 ± 2.1. Fifty-eight subjects (21%) were APOEε4 allele carriers. Concerning the dental check-up findings, the mean number of remaining teeth was 22.7 ± 7.5, 86 subjects (31%) had dental caries, and the CPI was ≥code 3 in 77 subjects (28%). Laboratory evaluations showed that *S. mutans, cnm*-positive *S. mutans*, and collagen binding activity were positive in 94%, 33%, and 25%, respectively, of the subjects. On brain MRI, CMBs were detected in 73 subjects (26%).

The age, BMI, frequency of subjects with a history of treatment for diabetes, frequency of those with drinking history, frequency of those with smoking history, number of years of schooling, frequency of those with dental caries, and MMSE score were significantly higher in males, and the frequency of those with *S. mutans* was significantly higher in females.

### Background of CMBs (+) and cognitive function

Table 2 compares the CMBs (+) (n = 73) and CMBs (−) (n = 206) groups. The CMBs (+) group showed a significantly higher BMI (*p* < 0.05) and significantly higher frequencies of those with a CPI ≥ code 3 (*p* < 0.05), *cnm*-positive *S. mutans*-isolated subjects (*p* < 0.01), DWMH ≥ grade 2 (*p* < 0.05) and collagen binding activity (+) subjects (*p* < 0.01). However, no significant difference was observed between the two groups in regard to hypertension, hyperlipidemia, diabetes, oral antiplatelet therapy and PVH grade. We did not see any relationship between CMBs and hs-CRP.

The type of CMBs were deep in 44 (60%), lobar in 20 (27%) and mixed in 9 (12%). We also evaluated the laterality of CMBs but found no right-left differences. On cognitive function evaluations, the CMBs (+) group showed significantly lower MMSE score and lower “Ta” score of the letter fluency tasks (*p* < 0.05 for both).

Table 3 shows the results of logistic regression analysis concerning the occurrence of CMBs. Crude analysis demonstrated a CPI ≥ code 3 (OR = 1.83, 95% CI: 1.03–3.25, *p* < 0.05) and collagen binding activity (+) (OR = 9.11, 95% CI: 4.94–16.8, *p* < 0.01) as significant risk factors for the occurrence of CMBs. In Model 2, in which all associated factors were applied, only collagen binding activity ≥10% was correlated with the risk of CMBs (OR = 14.3, 95% CI: 6.53–31.1, *p* < 0.01).

### Background and cognitive function of the *cnm*-positive *S. mutans* group and the collagen binding activity ≥10% group

Comparing the *cnm*-positive (n = 91) with the *cnm*-negative (n = 188) groups, no significant difference was observed in the clinical characteristics examined (Table 4). Cnm protein was collagen binding activity ≥10% in 78%. The distribution of CMBs was deep in 70%, lobar in 17% and mixed in 13% in the *cnm*-positive group with significant differences between the two groups. Deep CMBs were observed more frequently in the *cnm*-positive group, but lobar CMBs were observed more frequently in the *cnm*-negative group. Cognitive function evaluations showed no significant difference in the MMSE score, but the “Ka” score of the letter fluency tasks was significantly decreased in the *cnm*-positive group (*p* < 0.05 for “Ka”).

Comparing the collagen binding activity (+) (n = 71) with (−) (n = 208) groups, no marked difference was observed in clinical characteristics (Table 4). But the frequency of deep CMBs was significantly higher in the collagen binding activity ≥10% group (*p* < 0.01). Also, the percentage of subjects with dental caries was significantly higher (*p* < 0.01) in the collagen binding activity ≥10% group. Concerning cognitive functions, the “Ta” and “Ka” scores of the letter fluency tasks were significantly lower in the collagen binding activity ≥10% group (*p* < 0.05 for “Ta”, *p* < 0.01 for “Ka”).

## Discussion

We have previously reported that the collagen binding activity of Cnm protein produced by *cnm*-positive *S. mutans* is involved in the development of CMBs^13^. Our data showed that chronic presence of *cnm*-positive *S. mutans* in mice hematogenously induces cerebral hemorrhage through disruption of the blood-brain barrier^10^. In cerebral hemorrhage, endothelial cells of cerebral vessels are damaged, and collagen is exposed on the surface of the damaged blood vessel. Hemostasis caused by this exposed collagen is important as mechanism against cerebral hemorrhage, but, if *cnm*-positive *S. mutans* enters the blood from the mouth, its collagen binding activity may induce cerebral hemorrhage by adherence to and damage to the vascular endothelium^10^. However, whether these relationships between chronic *cnm*-positive *S. mutans* infection and cerebrovascular damage demonstrated in animals also applies to humans is unclear.

CMBs can be detected as punctate hypointensities by susceptibility-weighted imaging (SWI), a technique of brain MRI that is becoming increasingly prevalent, and pathological studies have confirmed that these hypointensities are hemorrhagic hemosiderin deposits^32^,^33^. CMBs have been observed in 4–6% of healthy individuals and 57–64% of patients with intracerebral hemorrhage^6^,^7^. CMBs were positive in 15% of the general population in the Rotterdam Scan Study, and their prevalence increased with age^34^. Yakushiji *et al*. reported that the prevalence of CMBs in over 70-year old healthy adults was around 15%, reflecting Japanese characteristics of CMBs^9^. CMBs positivity was relatively high at 26% in our study perhaps because of the high average age of the subjects (70 ± 6.1 years) and the high risk of hypertension, smoking and alcohol use. Deep CMBs were observed in 60% of our subjects. Bokura *et al*. reported the frequencies of deep, lobar, and mixed CMBs at 53%, 13%, and 34%, respectively, in an epidemiological survey of local residents^7^, and we obtained similar results. However, we noted no relationship between the prevalence of CMBs and hypertension or hyperlipidemia, which have been reported as risk factors of CMBs^6^,^7^,^35^,^36^. The mean age of our subjects was 70, which increases the prevalence of hypertension and CMBs.

We noted marked differences in the distribution of CMBs on brain MRI between *cnm*-positive and -negative groups, following the similar results in the collagen activity groups. It has been previously reported that the hs-CRP level, a biomarker of chronic inflammation, was high in the group with CMBs, suggesting their relationship with increased vulnerability of the vascular wall due to inflammation^37^. The percentage of patients with high hs-CRP was higher in the CMBs (+) group, but the difference was not significant. Nevertheless, the frequent occurrence of deep CMBs in patients with *cnm*-positive *S. mutans* strongly suggests an involvement of bacteria in secondary inflammation or injury of perforating arteries^38^. In subjects positive for *S. mutans* from the Japanese hospital-based cohort study^11^,^14^, the increased rate of collagen binding activity was similarly correlated with the number of deep CMBs, which is consistent with our results. A limitation of our work is that we did not measure inflammatory components other than hs-CRP, such as cytokines. Also, as a previous report suggested the relationship between the arterial stiffness and CMBs^39^, we performed evaluation of PWV, reflecting arteriosclerosis of peripheral vessels, but noted no significant difference.

Regarding the cognitive functional evaluations, the group of CMBs (+) showed low scores on the MMSE and letter fluency task starting with “Ta”, whereas it did not show statistical lower scores in letter fluency task starting with “Ka”, suggesting that it was not statistical significant because of lack of sufficient numbers. Another possibility is that the letter fluency task may be influenced by the affected cerebral location by CMBs in accordance with the functional representation of language.

Secondly, the infection rate of *cnm*-positive *S. mutans* was high in the CMBs (+) group. Also, collagen binding activity ≥10% showed the highest odds ratio for the occurrence of CMBs after adjustment for other factors. Thus, collagen binding activity of *cnm*-positive *S. mutans* was an independent risk factor of the occurrence of CMBs and in the CMBs (+) group, the percentage of patients with CPI ≥ code 3 was high. Comorbidities, such as gingivitis and periodontitis, increase vascular vulnerability, and entry of *cnm*-positive *S. mutans* into the blood, whoch may result in damage to cerebral vessels^40^. Also, the percentage of dental caries (+) patients was significantly higher in the collagen binding activity ≥10% group. Nomura *et al*. suggested a strong relationship between the occurrence of dental caries and the *cnm* gene^20^. Individuals with dental caries, often neglect appropriate brushing or dental treatment permitting proliferation of *cnm*-positive *S. mutans*, which binds to collagen, a component of dentin, increasing the percentage of collagen binding activity (+) in subjects in the dental caries (+) group^11^.

Thirdly, cognitive scores were decreased in the CMBs (+) group. Moreover, the scores of letter fluency tasks were significantly lower in the collagen binding activity ≥10% group. Yakushiji *et al*. reported that that CMBs cause cognitive impairment by damaging the basal ganglia- and the frontal-subcortical neuronal circuits^9^. Charidimou *et al*. observed that CMBs in the basal ganglia are often associated with microangiopathy, and are related to cognitive impairment in older people^41^,^42^. We suggest that collagen binding (+) *S. mutans* activates an indirect inflammation-mediated mechanism, which exacerbates microangiopathy in the region supplied by the perforator branches in the basal ganglia, thus increasing deep CMBs, and cognitive impairment. Moreover, because we confirmed that the MMSE scores were lower in females compared to males, and males had a smaller frequency of *S. mutans* positivity, we performed logistic analysis including sex as an adjusting factor. We also added education history as an adjusting factor. However, sex differences were not statistically significant.

We evaluated the relationships of PVH/DWMH, which reflect chronic ischemic changes of the brain, with *cnm*-positive *S. mutans* and the collagen binding activity. No significant relationship was noted in this study. In addition, APOE4 is widely known to be an allele that enhances cerebral vascular vulnerability and cognitive impairment^43^,^44^, and CMBs are observed more frequently in APOE4 carriers^45^,^46^. However, we found that the presence APOE4 was not significantly related to the presence of CMBs.

This study has the following limitations. Various confounding factors other than the items evaluated in this study may be involved in the associations involving cognitive impairment, CMBs and *cnm*-positive *S. mutans*. Because of the cross-sectional nature of this study, it was difficult to evaluate the relationships of brain MR or cognitive function with oral findings including *cnm*-positive *S. mutans* exposure. Longitudinal studies of the relationships of Cnm-positive *S. mutans* and CMBs will be of interest. Also we have not evaluated other factors related to oral hygiene besides the Cnm protein which may be conferring the occurrence of CMBs with cognitive decline. The data were obtained from a single center enrolling consecutive subjects responding to a random mailing in order to minimize the effect of individual selection bias. Although we used a random mailing to recruit subjects, some degree of selection bias was unavoidable. In addition, the number of subjects of this study was small and evaluation of a larger population is necessary. We understand that MMSE does not provide a sensitive examination of cognitive deficits. Further examination of executive functions will be advisable such as TMT (Trail Making Test) and the FAB (Frontal Assessment Battery). Furthermore, a comprehensive inflammatory profile was not obtained.

## Conclusions

The collagen binding activity of *cnm*-positive *S. mutans* was closely related to the occurrence of deep CMBs and may be a risk factor for cognitive impairment. These results are novel findings that link chronic oral infections with geriatric disorders, such as stroke and cognitive impairment, and suggest *cnm*-positive *S. mutans*, in particular, is a novel factor of cognitive impairment associated with CMBs. We now clarify in this population-based survey that the rate of *cnm*-positive *S. mutans* is also high in asymptomatic cerebral microbleeds, and is related to a decline of cognitive function. An intervention study focused on oral care and the microbiota in CMBs subjects will be of interest. These data further support the important influence of the oral microbiota on neurological disease and emphasize the importance of collaboration between dental and medical researchers.

## Additional Information

**How to cite this article**: Watanabe, I. *et al*. Oral Cnm-positive *Streptococcus Mutans* Expressing Collagen Binding Activity is a Risk Factor for Cerebral Microbleeds and Cognitive Impairment. *Sci. Rep.* **6**, 38561; doi: 10.1038/srep38561 (2016).

**Publisher's note:** Springer Nature remains neutral with regard to jurisdictional claims in published maps and institutional affiliations.

## Supplementary Material

Supplementary Materials

## Figures and Tables

**Table 1:** Characteristics by gender.

Variable	Males (n = 189)	Females (n = 90)	Total (n = 279)
	n (%)	n (%)	n (%)
Clinical characteristics			
Age (years) ± SD	70.2 ± 6.1	68.6 ± 6.0*	70.0 ± 6.1
Age (range)	54–87	60–89	54–89
BMI (kg/m^2^) ± SD	23.1 ± 2.7	22.0 ± 3.2**	22.7 ± 3.0
Hypertension	104 (55)	41 (46)	145 (52)
Hyperlipidemia	111 (59)	54 (60)	165 (59)
Diabetes mellitus	30 (16)	5 (5.6)*	35 (13)
History of stroke	4 (2.1)	1 (1.1)	5 (1.8)
History of cardiovascular disease	10 (5.3)	4 (4.4)	14 (5.0)
Antiplatelet therapy	23 (12)	6 (6.7)	29 (10)
Anticoagulant therapy	10 (5.3)	1 (1.1)	11 (3.9)
Drinking	138 (73)	43 (48)**	181 (65)
Smoking	27 (14)	4 (4.4)*	31 (11)
Education ≤12 years^a^	92 (49)	58 (66)**	150 (55)
baPWV (m/s) ± SD	1758 ± 228.2	1691 ± 333.4	1736 ± 304.6
hs-CRP ≥ 0.1 mg/dL	50 (27)	27 (30)	77 (28)
Dental check-up findings			
Number of remaining teeth ± SD	22.8 ± 7.7	22.5 ± 6.9	22.7 ± 7.5
Dental caries+	69 (37)	17 (19)**	86 (31)
CPI ≥ code 3	55 (29)	22 (24)	77 (28)
Laboratory evaluations			
APOE ε4 allele carrier^b^	35 (19)	23 (26)	58 (21)
*Streptococcus mutans*-isolated	171 (91)	90 (100)**	261 (94)
*cnm*-positive *S. mutans*-isolated	57 (30)	34 (38)	91 (33)
Collagen binding activity ≥10%	45 (24)	26 (29)	71 (25)
Brain MRI findings			
CMBs	49 (26)	24 (27)	73 (26)
PVH ≥ grade 2	24 (13)	11 (12)	35 (13)
DWMH ≥ grade 2	51 (27)	26 (29)	77 (28)
Cognitive functional evaluations			
MMSE (score) ± SD	28.6 ± 1.9	27.9 ± 2.5*	28.3 ± 2.1
Letter fluency tasks			
“Ta” (score) ± SD	8.3 ± 3.1	8.1 ± 3.9	8.2 ± 3.3
“Ka” (score) ± SD	9.9 ± 3.4	9.5 ± 3.3	9.8 ± 3.4

*T-tests *p* < 0.05 and Chi-square tests *p* < 0.05, **T-tests *p* < 0.01 and Chi-square tests *p* < 0.01.

^a^Data are missing for education (n = 4). ^b^Data are missing for APOE genotype (n = 7).

BMI; Body mass index, CPI; Community Periodontal Index, CMBs; Cerebral microbleeds.

PVH; Periventricular Hyperintensity, DWMH; Deep White Matter Hyperintensity, MMSE; Mini-Mental State Examination.

**Table 2:** Comparison of clinical characteristics between the cerebral microbleeds (+) group and (−) group.

Variable	Cerebral microbleeds	
	− (n = 206)	+ (n = 73)
	n (%)	n (%)
Clinical characteristics		
Age (years) ± SD	69.5 ± 5.9	70.2 ± 6.5
Age (range)	59–89	54–87
Sex (Male)	140 (68)	49 (67)
BMI (kg/m^2^) ± SD	22.5 ± 2.7	23.4 ± 3.6*
Hypertension	102 (50)	43 (59)
Hyperlipidemia	118 (57)	47 (64)
Diabetes mellitus	27 (13)	8 (11)
History of stroke	2 (1.0)	3 (4.1)
History of cardiovascular disease	8 (3.9)	6 (8.2)
Antiplatelet therapy	17 (8.3)	12 (16)
Anticoagulant therapy	7 (3.4)	4 (5.5)
Drinking	140 (68)	41 (56)
Smoking	26 (13)	5 (6.8)
Education ≤12 years^a^	108 (53)	42 (60)
baPWV (m/s) ± SD	1728 ± 306.9	1760 ± 298.9
hs-CRP ≥ 0.1 mg/dL	55 (27)	22 (30)
Dental check-up findings		
Number of remaining teeth ± SD	22.9 ± 7.2	22.3 ± 8.3
Dental caries+	60 (29)	26 (36)
CPI ≥ code 3	50 (24)	27 (37)*
Laboratory evaluations		
APOE ε4 allele carrier^b^	46 (23)	12 (17)
*Streptococcus mutans*-isolated	192 (93)	69 (95)
*cnm*-positive *S. mutans*-isolated	37 (18)	54 (74)**
Collagen binding activity ≥10%	28 (14)	43 (59)**
Brain MRI findings		
Distribution of CMBs		
Deep (%)	—	44 (60)
Lobar (%)	—	20 (27)
Mixed (%)	—	9 (12)
PVH ≥ grade 2	24 (12)	11 (15)
DWMH ≥ grade 2	50 (24)	27 (37)*
Cognitive functional evaluations		
MMSE (score) ± SD	28.5 ± 2.2	27.9 ± 2.0*
Letter fluency tasks		
“Ta” (score) ± SD	8.5 ± 3.5	7.6 ± 2.9*
“Ka” (score) ± SD	10.0 ± 3.5	9.2 ± 3.0

*T-tests *p* < 0.05 and Chi-square tests *p* < 0.05, **T-tests *p* < 0.01 and Chi-square tests *p* < 0.01.

^a^Data are missing for education (n = 4). ^b^Data are missing for APOE genotype (n = 7).

BMI; Body mass index, CPI; Community Periodontal Index, CMBs; Cerebral microbleeds.

PVH; Periventricular Hyperintensity, DWMH; Deep White Matter Hyperintensity, MMSE; Mini-Mental State Examination.

**Table 3:** Crude and adjusted odds ratio for the risk of cerebral microbleeds.

Variable	Crude	*p-value*	Model 1	*p-value*	Model 2	*p-value*
	OR (95% CI)^†^		OR (95% CI)^†^		OR (95% CI)^†^	
Age	1.02 (0.98–1.07)	0.37	1.02 (0.98–1.07)	0.36	1.05 (0.98–1.13)	0.15
Sex (Male)	0.96 (0.55–1.70)	0.90	0.93 (0.53–1.66)	0.81	0.90 (0.39–2.06)	0.80
BMI ≥25 kg/m^2^	1.42 (0.73–2.76)	0.30	1.02 (0.51–1.63)	0.76	1.24 (0.47–3.24)	0.66
Hypertension	1.46 (0.73–2.76)	0.17	1.47 (0.86–2.54)	0.16	1.36 (0.62–2.98)	0.44
Hyperlipidemia	1.35 (0.78–2.34)	0.29	1.38 (0.79–2.40)	0.26	1.59 (0.74–3.42)	0.23
diabetes mellitus	0.82 (0.35–1.89)	0.63	0.84 (0.36–1.96)	0.68	0.81 (0.26–2.56)	0.72
history of stroke	2.21 (0.74–6.62)	0.44	2.17 (0.72–6.49)	0.17	1.42 (0.31–6.46)	0.65
history of cardiovascular disease	4.37 (0.72–26.7)	0.11	3.97 (0.63–24.9)	0.14	2.94 (0.31–28.2)	0.35
Drinking	0.60 (0.35–1.04)	0.07	0.59 (0.34–1.05)	0.73	0.65 (0.30–1.38)	0.26
Smoking	0.51 (0.19–1.38)	0.18	0.54 (0.19–1.48)	0.23	1.07 (0.31–3.68)	0.92
antiplatelet therapy	2.19 (0.99–4.84)	0.53	2.12 (0.94–4.76)	0.07	2.16 (0.64–7.25)	0.21
anticoagulant therapy	1.65 (0.47–5.80)	0.44	1.63 (0.46–5.80)	0.45	2.77 (0.57–13.6)	0.21
Education ≤12 years	1.35 (0.78–2.34)	0.29	1.32 (0.75–2.31)	0.34	0.70 (0.33–1.47)	0.35
baPWV (m/s) ± SD	1.00 (0.99–1.00)	0.44	1.00 (0.99–1.00)	0.65	1.00 (0.99–1.00)	0.54
hs-CRP ≥0.1 mg/dL	1.18 (0.66–2.13)	0.57	1.15 (0.64–2.08)	0.64	0.72 (0.32–1.61)	0.43
Dental caries+	1.35 (0.77–2.37)	0.30	1.37 (0.77–2.44)	0.28	0.92 (0.42–2.01)	0.83
CPI ≥ code 3	1.83 (1.03–3.25)	**0.04**	1.84 (1.04–3.27)	**0.04**	2.01 (0.95–4.28)	0.07
APOE ε4 allele carrier	0.84 (0.40–1.75)	0.64	0.87 (0.48–1.58)	0.66	0.89 (0.33–2.38)	0.81
Collagen binding activity ≥10%	9.11 (4.94–16.8)	**<0.01**	9.68 (5.17–18.1)	**<0.01**	14.3 (6.53–31.1)	**<0.01**

^†^Odds ratio (OR), 95% confidence interval (CI), Bold values mean statistical significance at *p* < 0.05.

Model 1: adjusted by age and sex.

Model 2: adjusted by age, sex, BMI, hypertension, hyperlipidemia, diabetes mellitus, history of stroke and cardiovascular disease, alcohol consumption, cigarette smoking, antiplatelet therapy, anticoagulant therapy, education, baPWV, hs-CRP, dental caries, CPI, APOE ε4 allele carrier and collagen binding activity.

BMI; Body mass index, CPI; Community Periodontal Index.

**Table 4:** Comparison of characteristics between *cnm*-positive *Streptococcus mutans* (+) group and (−) group and background of collagen binding activity between the (+) group and the (−) group.

Variable	*cnm*-positive *S. mutans*-isolated		Collagen binding activity ≥10%	
	− (n = 188)	+ (n = 91)	− (n = 208)	+ (n = 71)
	n (%)	n (%)	n (%)	n (%)
Clinical characteristics				
Age (years) ± SD	70.1 ± 6.1	68.9 ± 5.9	70.0 ± 6.1	69.1 ± 6.0
Age (range)	54–89	60–87	54–89	60–87
Sex (Male)	132 (70)	57 (63)	144 (69)	45 (63)
BMI (kg/m^2^) ± SD	22.6 ± 2.5	23.0 ± 3.6	22.6 ± 2.7	23.0 ± 3.6
Hypertension	94 (50)	51 (56)	105 (51)	40 (56)
Hyperlipidemia	108 (57)	57 (63)	123 (59)	42 (59)
Diabetes mellitus	25 (13)	10 (11)	28 (14)	7 (9.9)
History of stroke	4 (2.1)	1 (1.1)	4 (1.9)	1 (1.4)
History of cardiovascular disease	7 (3.7)	7 (7.7)	9 (4.3)	5 (7.0)
Antiplatelet therapy	17 (9.0)	12 (13)	21 (10)	8 (11)
Anticoagulant therapy	8 (4.3)	3 (3.3)	9 (4.3)	2 (2.8)
Drinking	129 (69)	52 (57)	123 (59)	42 (59)
Smoking	24 (13)	7 (7.7)	12 (5.8)	4 (5.6)
Education ≤12 years^a^	102 (55)	48 (55)	112 (55)	38 (54)
baPWV (m/s) ± SD	1728 ± 292.5	1755 ± 329.5	1728 ± 298.3	1761 ± 323.6
hs-CRP ≥ 0.1 mg/dL	49 (26)	28 (31)	52 (25)	25 (35)
Dental check-up findings				
Number of remaining teeth ± SD	22.7 ± 7.5	22.8 ± 7.4	22.7 ± 7.5	22.8 ± 7.4
Dental caries+	52 (28)	34 (37)	52 (25)	34 (48)**
CPI ≥ code 3	48 (26)	29 (32)	56 (27)	21 (30)
Laboratory evaluations				
APOE ε4 allele carrier^b^	40 (22)	18 (20)	44 (22)	14 (20)
Collagen binding activity ≥10%	—	71 (78)	—	—
Brain MRI findings				
CMBs	19 (10)	54 (59)**	30 (14)	43 (61)**
Distribution of CMBs				
Deep (%)	6 (32)	38 (70)**	15 (50)	29 (67)**
Lobar (%)	11 (58)	9 (17)	13 (43)	7 (16)
Mixed (%)	2 (11)	7 (13)	2 (6.7)	7 (16)
PVH ≥ grade 2	24 (13)	11 (12)	26 (13)	9 (13)
DWMH ≥ grade 2	51 (27)	26 (29)	56 (27)	21 (30)
Cognitive functional evaluations				
MMSE (score) ± SD	28.4 ± 2.2	28.3 ± 2.0	28.3 ± 2.3	28.5 ± 1.8
Letter fluency tasks				
“Ta” (score) ± SD	8.4 ± 3.6	8.0 ± 2.8	8.5 ± 3.5	7.6 ± 2.8**
“Ka” (score) ± SD	10.1 ± 3.4	9.2 ± 3.2*	10.1 ± 3.4	8.9 ± 3.3**

*T-tests *p* < 0.05 and Chi-square tests *p* < 0.05, **T-tests *p* < 0.01 and Chi-square tests *p* < 0.01. ^a^Data are missing for education (n = 4).

^b^Data are missing for APOE genotype (n = 7). BMI; Body mass index, CPI; Community Periodontal Index, CMBs; Cerebral microbleeds.

PVH; Periventricular Hyperintensity, DWMH; Deep White Matter Hyperintensity, MMSE; Mini-Mental State Examination.
