# Supplementary material for: Oral Cnm-positive Streptococcus Mutans Expressing Collagen Binding Activity is a Risk Factor for Cerebral Microbleeds and Cognitive Impairment
Source: Sci Rep. 2016 Dec 9;6:38561. doi: 10.1038/srep38561 (PMC5146923; doi:10.1038/srep38561)
Supplement: Supplementary Materials [file srep38561-s1.doc]

**Oral Cnm*-*positive *Streptococcus Mutans* Expressing Collagen Binding Activity is a Risk Factor for Cerebral Microbleeds and Cognitive Impairment**

Isao Watanabe, Nagato　Kuriyama, Fumitaro Miyatani, Ryota Nomura, Shuhei Naka, Kazuhiko Nakano, Masafumi Ihara， Komei Iwai, Daisuke Matsui, Etsuko Ozaki, Teruhide Koyama, Masaru Nishigaki, Toshiro Yamamoto, Aiko Tamura, Toshiki Mizuno, Kentaro Akazawa, Akihiro Takada, Kazuo Takeda, Kei Yamada, Masanori Nakagawa, Tokutaro Tanaka, Narisato Kanamura, Robert P. Friedland and Yoshiyuki Watanabe

Supplemental materials:

Isolation of *S. mutans* and detection of the *cnm* gene by PCR

We sampled oral bacteria from all subjects by collecting ~ 5 ml of saliva in 50-ml centrifugal tubes (Corning®) and plaque around the teeth by scratching with a swab (Eiken Chemical Co., Ltd., Tokyo, Japan). The saliva sampled in the centrifugal tubes was stored at -20°C, and the swabs used for sampling of plaque were stored at 4°C. All measurements were performed within 3 days from sampling. We streaked 100 μl of saliva collected during a dental check-up on Mitis-Salivarius (MS) agar (Becton, Dickinson, Drive Franklin Lakes, NJ, USA). After 2 days anaerobic incubation at 37°C, we collected 5 bacterial colonies each under the microscope and cultured them in 10 ml of brain heart infusion broth (BD Difco™, Franklin Lakes, NJ, USA) for 1 more day. Then, we centrifuged the culture medium at 8,500 G for 5 minutes and added 250 μl of Glu-TE buffer and 60 μl of mutanolysin solution (*N*-acetylmuramidase SG 10 mg + MilliQ 5 ml). We mixed the medium, placed it in a warm water bath, and centrifuged it at 1,400 rpm for 1 minute. To extract DNA from the cells, we then added 600 μl of cell lysis solution (QIAGEN A, Germantown, Maryland, USA) to the Eppendorf tube containing the sample, incubated he mixture at 80°C for 5 minutes and 2 μl of RNAase A, and incubated the mixture again at 37°C for 30 minutes. To the Eppendorf tube, we added 200 μl of protein precipitation solution (QIAGEN Sciences) and mixed and centrifuged it at 1,450 rpm for 3 minutes. To the supernatant, we added 600 μl of isopropanol (Wako Pure Chemical Industries, Ltd., Osaka, Japan), centrifuged the mixture, and added 70% EtOH. Then, we added 100 μl of DNA hydration solution (QIAGEN Sciences), extracted 100 μl of DNA, and stored it.

Next, we suspended the DNA with an amplification reaction reagent, set the mixture in a thermal cycler, and performed PCR using PCR-related products that included high-performance PCR enzymes and real-time PCR equipment of Takara Bio Inc. (Shiga, Japan). We amplified DNA by PCR using My Cycler™ (BIO RAD Inc., Hercules, California, USA) over 30 cycles (94°C for 30 seconds, 60°C for 30 seconds, and 72°C for 2 minutes). We performed 1.5% agarose gel electrophoresis (Mupid®-2-plus: ADVANCE) using the sample obtained, detected a 100-bp single band, stained the agarose gel with ethidium bromide, checked the presence or absence of fluorescence of the band under UV irradiation, and evaluated the presence or absence of *S. mutans*.

Then, we determined the presence or absence of the *cnm* gene in the *S. mutans* obtained by PCR using *cnm*-specific primers. As described above, we amplified DNA, performed 0.7% agarose gel electrophoresis, and checked the presence or absence of the *cnm* gene according to whether or not a single band was detected at a 1 Kbp level.

Measurement of the Cnm protein-binding activity of *cnm*-positive *S. mutans*

We performed the collagen binding assay to measure the collagen binding activity of *cnm*-positive *S. mutans* detected in the subjects of this study. The assay was performed as reported previously^1^.

We smeared type I collagen that we prepared on a 96-well cell culture plate (Becton Dickinson, Ltd.) and stored it overnight at 4°C. We washed the plate 3 times with PBS, added a mixture of PBS and bovine serum albumin (BSA), and allowed the plate to stand at 37°C for 1.5 hours. After incubation, we washed the plate again with PBS mixed with 0.01%Tween-20 (Wako Pure Chemical Industries, Ltd.) and coated it with the collagen protein.

Also, we simultaneously incubated *S. mutans* isolated from the samples of the subjects in brain heart infusion broth (Becton, Dickinson), added it to each well of the above plate after adjusting its concentration using PBS to a fixed level, and co-cultured it at 37°C for 3 hours. Then, we washed the plate 3 times with PBS, added 100 μl of 25% formaldehyde, and allowed the plate to stand at room temperature for 30 minutes. We, thereafter, washed the plate again with PBS and added 100 μl of 0.05% crystal violet dye (Wako Pure Chemical Industries, Ltd.). After washing it out with PBS again, we added 100 μl of 7% acetic acid, and, after decomposition of the above dye, performed the binding assay at an optical density of 595 nm. The Cnm protein binding activity was expressed as the percentage of the activity of the sample relative to the activity of a standard strain (SA83/SA137). The Cnm protein binding activity was judged to be positive when this percentage was 10% or higher based on a previous report^1^.

Supplemental reference

1. Nomura, R. *et al*. Molecular and clinical analyses of the gene encoding the collagen-binding adhesin of *Streptococcus mutans*. *J Med Microbiol* **58**, 469-475 (2009).
